# Epitope Mapping and Computational Analysis of Anti-HPV16 E6 and E7 Antibodies in Single-Chain Format for Clinical Development as Antitumor Drugs

**DOI:** 10.3390/cancers12071803

**Published:** 2020-07-06

**Authors:** Carla Amici, Maria Gabriella Donà, Barbara Chirullo, Paola Di Bonito, Luisa Accardi

**Affiliations:** 1 Department of Biology, University of Rome Tor Vergata, 00133 Rome, Italy; carami371@gmail.com; 2 STI/HIV Unit, Istituto Dermatologico San Gallicano IRCCS, 00144 Rome, Italy; mariagabriella.dona@ifo.gov.it; 3 Department of Food Safety, Nutrition and Veterinary Public Health, Istituto Superiore di Sanità, 00161 Rome, Italy; barbara.chirullo@iss.it; 4 Department of Infectious Diseases, Istituto Superiore di Sanità, 00161 Rome, Italy; paola.dibonito@iss.it

**Keywords:** therapeutic antibodies, single-chain antibody fragments, HPV-associated cancer, clinical stage antibodies, Human Papillomavirus 16 E6 and E7 oncoproteins

## Abstract

Human Papillomavirus 16-associated cancer, affecting primarily the uterine cervix but, increasingly, other body districts, including the head–neck area, will long be a public health problem, despite there being a vaccine. Since the virus oncogenic activity is fully ascribed to the viral E6 and E7 oncoproteins, one of the therapeutic approaches for HPV16 cancer is based on specific antibodies in single-chain format targeting the E6/E7 activity. We analyzed the Complementarity Determining Regions, repositories of antigen-binding activity, of four anti-HPV16 E6 and -HPV16 E7 scFvs, to highlight possible conformity to biophysical properties, recognized to be advantageous for therapeutic use. By epitope mapping, using E7 mutants with amino acid deletions or variations, we investigated differences among the anti-16E7 scFvs in terms of antigen-binding capacity. We also performed computational analyses to determine whether length, total net charge, surface hydrophobicity, polarity and charge distribution conformed well to those of the antibodies that had already reached clinical use, through the application of developability guidelines derived from recent literature on clinical-stage antibodies, and the Therapeutic Antibodies Profiler software. Overall, our findings show that the scFvs investigated may represent valid candidates to be developed as therapeutic molecules for clinical use, and highlight characteristics that could be improved by molecular engineering.

## 1. Introduction

Recombinant monoclonal antibodies (mAbs) are among the classes of therapeutic drugs that convey most of the large funds from the biotechnology industry. Since 1986 up until to May 2020, the European Medicines Agency and the US Food and Drug Administration have approved ninety-four antibody therapies for the European or US market, while sixteen are under review [1] (Antibody Society. Approved antibodies. Available at https://www.antibodysociety.org/resources/approved-antibodies/).

Among the different formats of recombinant antibodies, single-chain variable antibody fragments (scFvs), consisting of the variable domains of the heavy (VH) and light (VL) immunoglobulin chains joined by a flexible linker, have probably the primacy of versatility. Indeed, they can be easily engineered by molecular biology techniques according to the purpose; e.g., grafted to different scaffolds, expressed as intracellular antibodies (intrabodies) by eukaryotic viral or non-viral vectors for delivery to tumor cells and tissues, or even administered as purified proteins directly to target cells [2,3]. A number of scFvs were utilized to specifically inhibit different protein functions and showed effective anti-tumor activity both in vitro and in vivo [3,4,5].

Among the over 200 Human Papillomavirus (HPV) genotypes discovered in humans (PaVe Database) [6], only twelve to fourteen are defined as high risk (HR) and are causally related to virtually all tumor lesions of the cervix, a high proportion of squamous cell carcinomas (SCC) in the ano-genital region and an increasing number of those in the head and neck area (HNSCC) [7,8]. Among the HR HPVs, HPV16 is the most represented in all body districts, and almost the only genotype present in the HPV-related oropharyngeal SCC, which comprise 30% of the total HNSCC [9].

The whole pro-oncogenic activity of the HR HPVs is in charge of the E6 and E7 oncoproteins, which are the first (Early) viral proteins transcribed from the same mRNA during infection. E7 is a 98 amino acid (aa) phosphoprotein comprising three conserved regions: CR1 (aa 2 to 15), CR2 (aa 16 to 37) and CR3 (aa 38 to 98) [10,11]. The CR3 region at the C-terminus is highly structured and comprises a zinc finger domain (aa 58–61 and 91–94) proposed to be involved in protein oligomerization [12]. The E7 N-terminus, which includes residues 1–40 (CR1/CR2), is instead unstructured and represents an intrinsically disordered region determining the protein plasticity, where the capacity to assume different conformations determines the E7 stability and correlates with its capacity to bind to different targets, involved or not in the transforming activity [13]. In particular, aa residues 21–26 in the CR2 region are responsible for the binding to the pRb tumor suppressor, crucial for the transforming activity. Furthermore, cysteine (C) at position 24 is a redox center that, under oxidative stress in HPV-transformed cells, undergoes glutathionilation, hindering pRb binding. This region also binds to TMEM173/STING, causing the inhibition of the antiviral response [14]. Instead CR1 has a role in transformation unrelated to the interaction with pRb, where the deletion of positions 6–10 (PTLHE) inactivates the E7 transforming activity but does not affect its transactivating capacity [15].

E6 is a 158 aa protein characterized by the presence of two Zinc finger domains (residues 37–73 and 110–146) linked by a LXXLL binding motif responsible for the proteasome-mediated degradation of a number of cellular substrates, including the p53 tumor suppressor [16]. The PDZ sequences (residues 156–158) at the C-terminus also participate in the E6 oncogenicity by the interaction with proteins implicated in cellular adhesion and polarity control [17].

The continuous expression of E6 and E7 during a persistent infection with HR-HPV can induce, over the years, the development of tumors in which the two oncoproteins represent the Tumor Associate Antigens (TAA) [18,19]. Therefore, targeting E6 and E7 offers the possibility of counteracting the development and/or progression of pre-tumor and tumor lesions for which the current HPV vaccine, effective in preventing infection, is not useful, since it has not been designed for therapeutic purposes. That early, HPV-induced pre-neoplastic lesions are localized in a limited area, which is an additional advantage for therapy, as it allows for local treatment [20]. This approach may also represent a therapeutic option for HPV-positive oropharyngeal SCC, which presently has poor disease outcome [21]. At present, the available prophylactic vaccines are not approved for the prevention of this tumor.

In the past few years, we isolated four scFvs against the E7 (scFv9, scFv32, scFv43 and scFv51) and one scFv against the E6 (scFvI7) oncoprotein of HPV16. The scFv43 was modified by site-directed mutagenesis to increase its stability [22], and the resulting scFv43M2 used thereafter. ScFv43M2 fused with the SEKDEL signal for retention in the endoplasmic reticulum, and scFvI7 fused with the nuclear localization signal (NLS), expressed as intrabodies, demonstrating a strong antiproliferative activity in HPV16–positive cells in vitro as well as antitumor efficacy in preclinical models in vivo [23,24,25].

Here, we compared the reactivity of the anti-HPV16 E6 (16E6) and -HPV16 E7 (16E7) scFvs against their targets, and identified the E7 regions recognized by the different anti-16E7 scFvs. 

It is well known that the Complementarity Determining Regions (CDRs) present in the antibody VH and VL domains are responsible for various antibody properties in addition to the ability of antigen-binding, including solubility and stability.

Many studies analyzed the CDRs charge of therapeutic antibodies in an attempt to decipher a common signature and predict the proper functioning of new antibody candidates. One of these studies analyzed more than 100 clinical-stage antibodies and found a strong correlation between the negative net charge of CDRs on the one hand, and a high binding specificity, good solubility, efficient refolding and the low self-association and aggregation of the antibody molecule on the other hand [26]. In particular, it was reported that negative charged residues, such as aspartate (D) and glutamine (E) positively correlate with the probability of favorable scFv properties, whereas positive charged residues, such as arginine (R) and lysine (K), show a negative correlation. An exception to this rule is represented by the hydrophobic leucine (L) residues, a high number of which correlates with a lower specificity of binding and poor biophysical properties. 

A different study analyzed a large set of post-phase I clinical-stage antibody therapeutics (CSTs) and identified shared CDR characteristics indicative of a possible development as antibodies for clinical use, leading to the implementation of the Therapeutic Antibody Profiler (TAP) software [27]. TAP allows for the comparative analysis of new antibody candidates with CSTs by analyzing five key parameters: (i) total CDR length; (ii) extent and magnitude of surface hydrophobicity (Patches of Surface Hydrophobicity Metric, PSH); (iii) Patches of Positive Charge Metric (PPC); (iv) Patches of Negative Charge Metric (PNC); (v) asymmetry in the net heavy- and light-chain surface changes (Structural Fv Charge Symmetry Parameter, SFvCSP), responsible for high viscosity. 

Bearing in mind the possible use of our scFvs for the treatment of HPV16 lesions, we evaluated the scFv specificity based on the charge of the aa residues that form their CDRs, and investigated by TAP the properties potentially favoring their therapeutic development.

## 2. Results

### 2.1. CDRs aa Sequences of the Anti-16E6 and -16E7 ScFvs

Sequence analysis of the four anti-16E7 scFv9, scFv32, scFv43 and scFv51 confirmed the VH origin from the DP47 germline gene. As far as it concerns the VL origin, scFv43 derives from the DPK22, while scFv9, scFv32 and scFv51 derive from the DPL16 germline gene [28]. The diversity of the ETH-2 library (10^8^ clones) used to select these antibodies is entirely up to the VH and VL CDR3 that have been modified by the random mutagenesis of selected nucleotide positions [28].

The CDRs of the VL and VH domains of the anti-16E6 scFvI7 were compared by sequence alignment to the IgG database using the IgG blast tool. Of note, due to the library construction, the VH and VL in the anti-16E6 scFv are in reverse order compared to the anti-16E7 scFvs, with the VL deriving from murine IgKV6-23 germline gene located at the N-terminus upstream of the linker, and the VH deriving from IGHV1S81 located at the C-terminus, downstream thereof.

The aa compositions of the CDRs are shown in Table 1. ScFv43M2 is indicated in place of the originally selected scFv43. The two scFvs have the same specificity but scFv43M2 carries an aa variation from T to M at position VH34 (numbering according to Kabat) [29]. The change, obtained by site-directed mutagenesis, re-established the original sequence of the germline gene according to the VH consensus, and successfully improved the scFv stability [22].

### 2.2. Analysis of the Anti-16E6 and Anti-16E7 ScFvs Binding Activity

**Figure 1:**
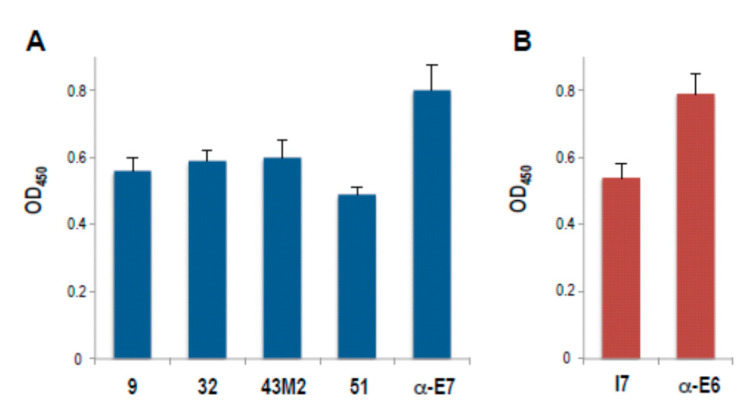
Characterization of the anti-16E7 and anti-16E6 scFvs reactivity against the respective recombinant oncoproteins by ELISA. Data represent the mean ± SD of samples in quadruplicate from a representative experiment of three with similar results. (**A**) the anti-16E7 scFv9 (9), scFv32 (32), scFv43M2 (43M2), scFv51 (51) and (**B**) the anti-16E6 scFvI7 (I7) are indicated. Anti-16E7 polyclonal Ab and anti-16E6 mAb were used as positive controls.

The reactivity of all the anti-16 E7 scFvs was confirmed by a comparative ELISA using the purified scFvs proteins against the recombinant antigen, as reported in the Materials and Methods. As shown in Figure 1A, all the anti-16E7 scFvs were able to recognize their antigen. Since reactivity against multiple nonspecific antigens was observed for scFv9 (personal communication), the features of this antibody were not further investigated. ScFvI7 and an anti-16E6 polyclonal Ab were included in the analysis as internal controls, since their anti-E6 binding activity had been previously demonstrated, as shown in Figure 1B [25].

**Figure 2:**
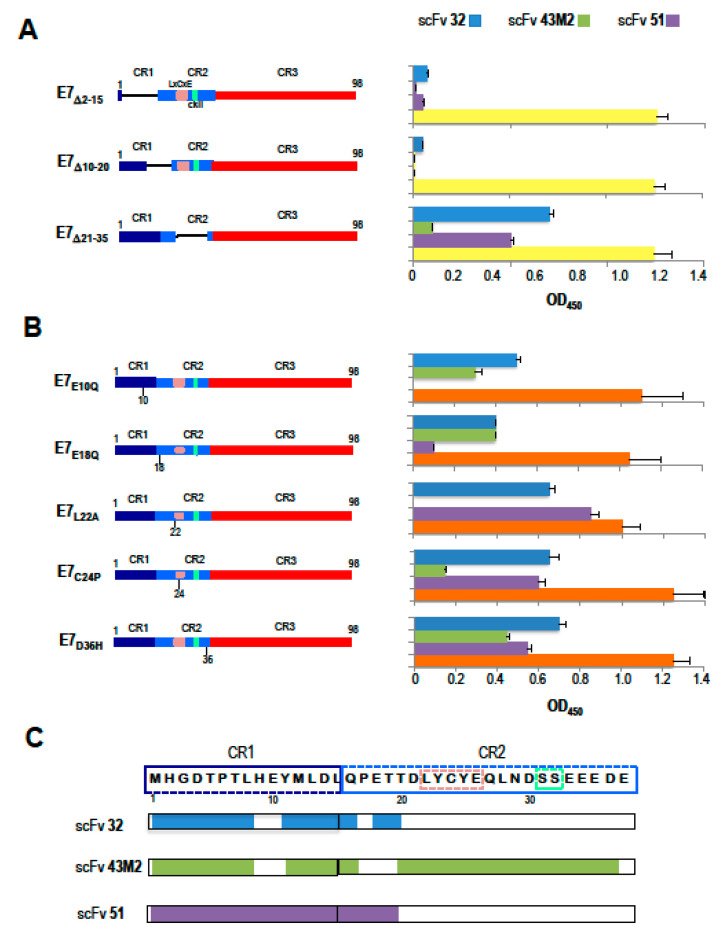
Epitope mapping of anti-E7 scFvs binding site on the 16E7 oncoprotein. (**A**) Schematic representation of 16E7 deletion mutants with the deleted amino acids, indicated by Δ, is shown on the left. The conserved CR1, CR2 and CR3 regions, the conserved LxCxE motif responsible for pRb binding and the casein kinase II (CKII) phosphorylation site in the CR2, are also indicated. The bars on the right represent the reactivity in ELISA, of scFv32 (light blue), scFv43M2 (green), scFv51 (violet) and in-house-made anti-E7 mouse polyclonal antibody (yellow), against each mutant. Data are expressed as the mean ± SD of three independent experiments, each performed in triplicate. (**B**) Schematic representation of the 16E7 mutants carrying single aa variations is shown on the left. E7_E10Q_ has a variation from glutamic acid (E) to glutamine (Q) at position 10, and the same change is present in E7_EQ18_ at position 18, while E7_L22A_ has a variation from leucine (L) to alanine (A) at position 22. E7_C24P_ has proline (P) instead of cysteine (C) at position 24, while E7_D36H_ has histidine (H) instead of aspartic acid (D) at position 36. The results of the ELISA performed with the same mutants are shown on the right. The color code for scFvs is the same as in panel A. The reactivity of the anti-E7 mAb is indicated in orange. Values are the mean ± SD of four independent experiments, each performed in triplicate. (**C**) The aa sequence of the E7 N-terminal region. CR1 and CR2 sequences, as well as the pRb binding and the CKII-phosphorylation sites in the CR2, are delineated by dashed line boxes with color codes, as in panels A and B. The E7 regions, bound by the scFvs indicated on the left, are highlighted by the respective color codes.

We previously demonstrated that scFv43M2 and scFv51 specifically bind to the E7 N-terminus (residues 1–54) region that retains important functions, including the transforming activity. From the same studies, we also know that scFv43M2 and scFv51 bind to different E7 epitopes [30]. Instead, we had no indication of the scFv32 binding regions on E7. To map the binding sites of scFv32, scFv43M2 and scFv51 on E7 in detail, as antigens we used a number of GST-tagged E7 proteins carrying either deletions or single aa variations previously designed to map the contribution of specific domains of the E7 gene product in the transcriptional trans-activation and cellular transformation functions [31]. The E7 mutants with aa deletions or variations are represented in Figure 2A,B, respectively.

The ELISA results demonstrated that scFv32 and scFv51 did not recognize E7_Δ2–15_ and E7_Δ10–20_, while they were still able to recognize E7_Δ21–35_. This means that the E7 region, comprised between aa at positions 2 and 20, which includes CR1 and part of CR2, is critical for the binding of scFv32 and scFv51 to E7, while the adjacent region 21–35 is dispensable. Therefore, the two scFvs have a similar valence with regard to the binding of their target E7. On the other hand, scFv43M2 binding was greatly reduced using E7_Δ21–35_ and absent using E7_Δ2–15_ and E7_Δ10–20_, as shown in Figure 2A, indicating that this scFv recognizes CR1 and almost the whole CR2. Western blot analysis confirmed the ELISA results (personal communication).

The E7 proteins carrying single aa variations allowed to better define the epitope binding sites on E7. As shown in Figure 2B, scFv32 and scFv43M2 retained the binding to E7_E10Q_ and E7_E18Q_ mutants while both variations abrogated the binding of scFv51. The A22L and C24P variations abolished the scFv43M2 binding to E7, while they were irrelevant for the scFv32 and scFv51 binding. Lastly, all the scFvs could bind to the E7_D36H_ mutant. Again, the ELISA results were confirmed by Western blot analysis (personal communication).

Taken together, these results indicate that scFv32 is able to recognize an E7 domain, comprised between positions 1 and 20, which includes CR1 and part of CR2, as well as scFv51, which, however, seems to be affected by the E18Q variation. On the other side, scFv43M2, not recognizing the E7 with A22L and C24P variations, nor the E7_Δ21–35_ mutant, binds to a wider region, comprising CR1 and CR2, and confirms the potential ability to interfere directly with the pRb-E7 interaction. A schematic representation of the scFvs binding sites on the E7 region, including the first N-terminal 37 aa, based on the results above described, is shown in Figure 2C.

**Figure 3:**
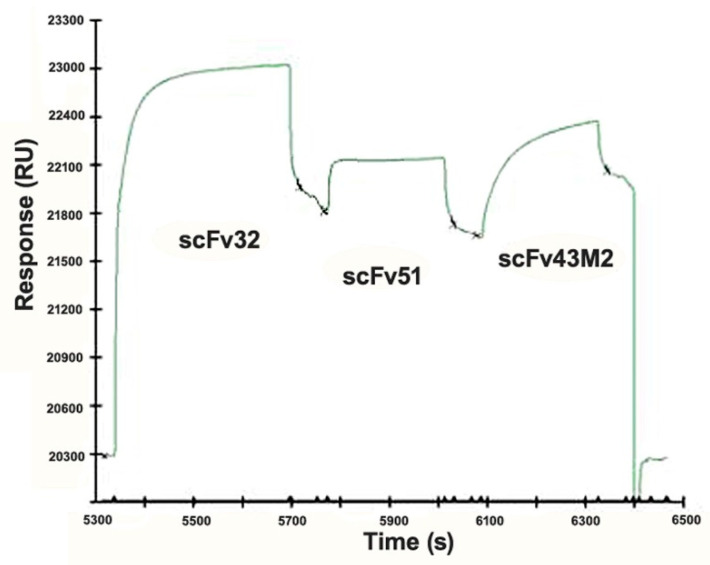
Surface Plasmon Resonance (SPR) analysis of the anti-16E7 scFv32, scFv43M2 and scFv51 binding to the 16E7 protein. The sensorgram shows the relative binding of the three scFvs to the recombinant 16E7 immobilized on the sensor chip. ScFv32 at saturating concentration was injected, followed by scFv51 and then by scFv43M2.

To better define the binding differences among the three anti-16E7 scFvs, we analyzed, by Surface Plasmon Resonance (SPR), the ability of the purified scFv proteins to bind to the recombinant wild type 16E7, covalently immobilized on the surface of a CM5 sensor chip. This was accomplished in a competition test by studying the binding interference among the three scFvs. As shown in Figure 3, the saturation of E7 with scFv32 was able to partially hamper the scFv51 binding, indicating that the E7-binding regions of the two scFvs were at least partly overlapped. ScFv43M2 injected after scFv51 was still able to bind to the sensor chip, showing that its ability to bind the E7 is not hampered by the previously injected scFvs. From this result we can also deduce that scFv43M2 has binding epitopes different from both scFv32 and scFv51.

### 2.3. Computational Analyses of The Anti-16E7 and -16E6 Scfvs aa Sequences

To evaluate the potential therapeutic efficacy of our anti-16E6 and -16E7 scFvs, we first calculated the CDR3 net charge at neutral pH, based on the presence of positively and negatively charged aa residues as predictors of antibody specificity [25]. As reported in Table 2, both the VH and VL CDR3s were in the range of −1 to +3.1. By adding the net charges of the VH and VL CDR3, most anti-16E7 scFvs have a slightly positive net charge in the range of +1 to +2, with the exception of scFv43M2 (+4.1), while the anti-16E6 scFv has slightly negative CDR3 net charge (−1).

Since it has been reported that the net charge of the entire set of six CDRs is a better predictor of antibody specificity than the CDR3 charge, or even of the entire scFv, we then calculated the theoretical total CDR net charge at neutral pH, as shown in Table 2. All the anti-16E7 scFvs showed a positive total net charge of around +4.0, different from the anti-16E6 scFvI7 that had a slightly negative total CDRs net charge (−0.9). Furthermore, since non polar or polar non charged aa residues in CDRs were also reported to contribute positively or negatively to specificity [26], we calculated their number in our scFvs and found that the CDR aa residues positively related to specificity are 1.4 to 1.8 fold those negatively related.

We then analyzed our scFvs by TAP software, and the results, reported in Table 3, show the average values obtained for the properties analyzed. We did not include scFv9 in this analysis because of its abovementioned nonspecific reactivity, nor did we include scFvI7. Indeed, due to the murine scaffold, in the case of the therapeutic development of scFvI7, it is necessary to graft the CDRs into a scaffold of human origin.

The anti-16E7 scFv32 and scFv51 display values in full accordance with CSTs, whereas the anti-16E7 scFv43M2 has suboptimal PPC, with this value falling in the extreme 5% of the CSTs distribution.

## 3. Discussion

Targeting the HPV oncoprotein activity has been explored in a number of therapeutic approaches for HPV-associated tumors, mainly because it warrants high precision and specificity due to the oncoprotein key role in HPV tumor onset and progression [33]. Because of the dominant role played by HPV16 in HPV-associated cancers, most of the studies have focused on the oncoproteins encoded by this genotype.

Our approach aimed at hampering the activity of the 16E6 and 16E7 oncoproteins through antibodies in single-chain format (scFvs), which, due to their flexible format, can be tailored and improved according to their needs.

In our laboratory, we isolated a number of anti-16E7 scFvs, namely scFv9, scFv32, scFv43M2 and scFv51, and the anti-16E6 scFvI7. All the scFvs exhibit a good reactivity for their respective antigens, as shown in Figure 1.

It is well known that the 16E7 is a multi-functional protein that possesses a mosaic of oncogenic activities whose partial or total ablation has therapeutic implications. Since the different scFvs selected, all recognizing the E7 N-terminus [30], can bind to different epitopes and potentially interfere with different functions of this protein, to map in detail such epitopes, we took advantage of a series of E7 mutants carrying either aa deletions or single aa variations, that we used in immunoassays. The observation that neither scFv32 nor scFv51 were able to recognize the E7_Δ2–15_ and E7_Δ10–20_ deletion mutants made it clear that both scFvs recognize the E7 N-terminal region, including the CR1 domain and some amino acids of CR2, critical for the trans-activating and anti-apoptotic activity of the oncoprotein [10]. In the meantime, they both recognize E7_Δ21-35_, and their binding is not affected by variations at positions 22 and 24, indicating that their function is not directly involved in hampering the E7 binding to pRb. On the other hand, scFv32 was able to bind to the E7_E10Q_ and E7_E18Q_ mutants, suggesting that scFv32 binding is not affected by variation from a negatively charged residue (E) to a non-charged residue (Q). ScFv51 behaves differently, since both variations at positions 10 and 18 abolish its binding to E7. The scFv43M2 binding to E7 is instead hampered by variations at positions 22 and 24, confirming the already demonstrated interference of this antibody with the E7 binding to the pRb tumor suppressor [30]. Finally, all the scFvs can bind to E7_D36H_, indicating that the CKII serine-specific kinase region [34,35] is not involved in the anti-E7 scFv activities. The results obtained with Surface Plasmon Resonance confirmed the partial overlapping of the scFv32 and scFv51 binding sites, and also that they differ from the epitopes bound by scFv43M2.

Studies on antibody optimization for therapeutic use have long found that the CDR charge is relevant for the antibody effectiveness, in influencing properties, such as folding stability, solubility and pharmacokinetics [36]. Recently, the theoretical CDR net charge was demonstrated to be a strong predictor of the antibody specificity, where negatively charged CDR3 and total CDRs were found to confer antibodies a higher specificity with respect to the positively charged CDRs [26]. When analyzing our anti-E6 and -E7 scFvs in terms of CDR3 and total CDR net charge, to identify which of them have promising characteristics for therapeutic development, we found values of CDR net charge of around +4.0, except for the anti-16E6 scFv, which exhibits a slightly negative charge and values ranging from −1.0 to + 3.0 for the VH and VL CDR3 charges.

In their paper, Rabia et al. analyzed 137 antibodies in clinical stage to identify values of CDR charges optimal for antibody specificity and biophysical properties. Of note, even though our scFvs have values of CDR charge not fully matching the optimal values, 20–35% (depending on the number of parameters considered) of the antibodies analyzed by Rabia with CDR net charge ≥ + 2.0 had favorable biophysical properties [26].

Furthermore, when we considered other aa residues—uncharged polar and non-polar—that can contribute negatively or positively to CDR specificity [26], we found that the number of residues with positive correlation far exceeds that of residues with negative correlation. Additionally, the low number of L residues in the CDRs of scFv32 and scFv51, and the absence of L in scFv43M2 and scFvI7, are not in contrast with a specific interaction between the scFvs and their targets. We also compared our scFvs to accredited therapeutic antibodies by using the recently developed Therapeutic Antibodies Profiler (TAP) [27]. TAP considers both the sequence and structural properties of a large set of CSTs and establishes threshold values for five parameters examined so that new candidate molecules can be scored to highlight potential weaknesses, discouraging therapeutic development. TAP analysis of our scFvs showed a good agreement in general with the optimal values for all the parameters, with only few values falling in the extreme 5% of the CSTs distribution. However, it is important to note that, as far as it concerns the properties considered, all our scFvs show values falling within the range of values belonging to the CSTs analyzed by the TAP software [27]. Additionally, scFv43M2, which a displays suboptimal PPC score, has already shown an effective antiproliferative and antitumor activity [24,25]. In this regard, it should be remembered that TAP analysis allows a theoretical prediction based on the assumption that antibodies that have reached phase I clinical trials have characteristics favorable to therapeutic development, but it does not consider antibody features, such as immunogenicity or stability, which are equally important for therapeutic applications. In fact, few antibodies in clinical use have parameters which do conform to the desirable values according to TAP.

In summary, our anti-E7 scFvs scFv32 and 51, although differing in the CDR3 sequences, bind to an almost identical region of E7, have overlapping characteristics with regard to their E7 binding capacity and exhibit similar values of CDR3, total CDR net charge and TAP analyses. Hence, from the point of view of therapeutic development, these two scFvs can be considered equivalent, with an advantage for scFv51 because of its anti-proliferative activity, already demonstrated. ScFv43M2 binds instead to a different region of E7, including the pRb-binding region, which is critical for the protein transforming activity, and was characterized in previous studies showing its effectiveness as an antitumor molecule. Therefore, it is worth pursuing future research regarding the clinical application of these antibodies, alone or in combination.

We believe that the information obtained by these analyses could represent a starting point to optimize the properties of the scFvs and to support decisions on their therapeutic development. Indeed, small variations in the aa sequence can have an impact on the antibody biophysical characteristics, as demonstrated by the increase in scFv43 half-life obtained by site-directed mutagenesis [22].

Once particularly favorable CDRs have been identified in an antibody framework, the cassettes, including the VH and VL CDRs, can be grafted onto different antibody scaffolds, as needed [37].

In addition, the information acquired could help identify scFvs to be used in combination or for the construction of bi-specific scFvs, in order to enhance the antitumor effect by targeting different epitopes of the same oncoprotein, or both the E6 and E7 proteins at the same time.

## 4. Materials and Methods

### 4.1. ETH-2 and SPLINT Libraries of Recombinant Single-Chain Antibodies

ETH-2 is a phage display library of human recombinant antibodies in single-chain format, consisting of a single polypeptide chain that includes an antibody VH joined by a flexible polypeptide linker to a VL domain [28]. Only DPK-22 and DPL-16 for the light chain, and DP-47 for the heavy chain, were used as germ-line genes for the library construction [28]. To create a large repertoire of antibodies, random loops of 4-5-6 amino acids were appended to position 95 of the VH CDR3, whereas six aa positions were altered in the VL CDR3. The library was cloned in the NcoI and NotI restriction sites of the pDN332 phagemid vector, containing the M13 origin of replication, the *E. coli* origin of replication, an Amp resistance, a peptide leader and a lac-Z promotor [28]. All scFvs expressed using this vector were fused with a FLAG-tag and a 6xHistidine-tag (His-tag) at the C-terminus.

The Single Pot Library of Intracellular antibodies (SPLINT) is a murine naıve library of scFv fragments expressed in the yeast cytoplasm. SPLINT construction was detailed elsewhere [38].

### 4.2. ScFv Selection

The selection of the anti-16E7 scFvs from the ETH-2 library was described in Accardi et al. [23]. Three rounds of panning in solution were carried out against the biotinylated recombinant His-E7 protein, according to Pini et al. [28]. The selected anti-E7 scFvs are cloned in the pDN332 phagermid vector under the lac z-promoter control.

The selection of the anti-16E6 scFvI7 from the murine SPLINT is described elsewhere [25]. Briefly, the SPLINT was transformed in the L40 yeast containing 16E6-expressing bait. After two yeast screenings for LacZ activity and histidine prototrophy, one positive clone, specifically interacting with 16E6 bait and not interacting with lamin bait used as an irrelevant antigen, was identified among transformants by Intracellular Antibody Capture Technology (IACT) [38].

### 4.3. Sequencing

For the sequence analysis of the CDR3 regions responsible for the diversity of the anti-16E7 antibodies, two primers were used, specifically:

DP47CDR2back (priming in the VH germline gene, before the VH CDR3)

5′-TAC TAC GCA GAC TCC GTC AAC-3′;

fdseq1 (priming at the beginning of the phage gene III, which is located downstream of the scFv sequence);

5′-GAA TTT TCT GTA TGA GG-3′.

Other primers designed to cover the whole scFv sequences were used, specifically:

PelBback (priming on the PelB leader, which is located upstream of the scFv sequence);

5′-AGC CGC TGG ATT GTT ATT AC-3′

C3 (closer to the VH CDR3):

5′-TACTACGCAGACTCCGTGAAG-3′

GVL (closer to the VLCDR3):

5′-CTCTCCTGCAGGGCCAG-3′.

The scFvI7 cloned in scFvExpress was sequenced using the following primers to cover both strands:

ScFvExRev

5′-GAG GGG CAA ACA ACA GAT GG-3′;

antiE6seqDir

5′-GTC CCT GAT CGC TTC ACA GG-3′;

antiE6seqRev

5′-CCC AGA ACC GCT GGT CGA CC-3′.

Sequence alignments to the NCBI database were carried out using Immunoglobulin BLAST.

### 4.4. Plasmids for Protein Expression

The anti-16E7 scFvs were all inserted in the pDN332 phagemid, also allowing expression in the prokaryotic systems [28]. Interestingly, the coding sequences of the anti-16E6 scFvI7, which had been selected as an intrabody, were subcloned into the scFvExCyto-SV5 eukaryotic vector for expression in cell cytoplasm, and into the pQE30 prokaryotic vector for protein expression [25,39].

Full-length 16E6 and 16E7, fused to a 6-His Tag tail, were constructed by cloning in the pQE-30 vectors (Qiagen, Chatsworth, Ca), as described in Di Bonito et al. 2006 [40] and Accardi et al. 2005 [23]. The JM109 strain of *E. coli* was transformed with the recombinant pQE-30 plasmids.

The recombinant plasmids expressing mutant E7 proteins carrying specific deletions or aa variations (kindly provided by David Pim, ICGEB, Trieste, Italy) were cloned in the pGEX-2T vector (Sigma-Aldrich, Italy) and expressed as Glutathione-transferase (GST)-fusion proteins in the *E. coli* DH5a strain.

### 4.5. Protein Purification

The extraction of scFvs and of the E6 and E7 proteins was performed from the respective transformed bacteria, and the proteins were purified using protein A-Sepharose CL-4B agarose beads (Amersham Biosciences) for the scFvs, and Ni-NTA agarose beads (Qiagen) for the oncoproteins, as previously reported [22,23,39,40].

The purity of the proteins was evaluated by Coomassie Blue Staining after SDS-PAGE, and the protein concentration was determined by Bradford assay (BioRad, Italy).

The E7 mutants were purified by affinity chromatography using GST-Sepharose (Invitrogen-ThermoFisher Scientific, Waltham, MA, USA) as described in Accardi et al. [30].

### 4.6. ScFv Reactivity

The reactivity of the purified scFvs (3 μg/mL) towards the recombinant His-E7 or His-E6 proteins (0.3 μg/well), immobilized onto microtiter 96-well plates in carbonate buffer (pH 9.4) at 4° O/N, was tested in ELISA. The experimental conditions used in this assay are different from those used for scFv selection by Phage Display, where the biotinylated His-E7 was employed for panning in solution. The scFv binding was detected by incubation with mouse anti-Flag M2 monoclonal antibody (mAb) (2 μg/mL) (Sigma, St. Louis, MO, USA), which recognizes the FLAG-tag at the scFv C terminus. Anti-E7 polyclonal Ab produced in mice in our laboratory and anti-E6 mAb (Invitrogen-ThermoFisher Scientific, Europe), diluted 1:500, were used as positive controls [40]. After extensive washing, the immune complexes were revealed by goat anti-mouse Horseradish peroxidase-conjugated (GAM-HRP) IgG (Amresco, Krackeler Scientific, Albany, NY, USA) and by using the TMB substrate kit for peroxidase (Vector Laboratories, Inc., Burlingame, CA, USA) for colorimetric evaluation. OD was measured at 450 nm in a microtiter plate reader (iMark, Bio-Rad, CA).

### 4.7. Epitope Mapping of the Anti-16E7 ScFvs

Analysis by ELISA was performed using a number of GST-tagged E7 proteins carrying either a deletion of aa stretches or a single aa variation as coating antigens (kindly provided by Dr David Pim). In particular, the E7_Δ2–15_ and E7_Δ10–20_ deletion mutants lack two different but overlapping regions, the first one belonging to CR1 and the second one also including few amino acids of CR2, whereas E7_Δ21–35_ maps in CR2 include the aa 20–29 involved in the binding of pRb and TMEM173/STING. As far as regards the single aa variations, both E7_E10Q_ and E7_E18Q_ have a variation from the negative charged glutamic acid (E) to the polar non-charged glutamine (Q); E7_L22A_ has a variation from the hydrophobic leucine (L) to a small, less hydrophobic alanine (A); E7_C24P_ has a variation from the reactive, sulfur-containing cysteine (C) to the cyclic non-polar proline (P); E7_D36H_ has a variation from the negative charged aspartic acid (D) to the positively charged histidine (H). The scFvs in immune-complexes were detected as described above and according to the protocol reported elsewhere [30]. The non-specific signal of the scFvs bound to the recombinant GST protein (Kerafast, Boston, USA) was used as a cut-off value.

Western blotting analysis was performed to confirm the ELISA results. Each mutant E7 protein was separated on 15% SDS-PAGE and electro-blotted with semidry apparatus (Bio-Rad, CA) onto PVDF Immobilon-P membrane (Millipore). The membrane was then cut in strips (1µg of protein/strip) and incubated with the purified scFvs at 2 µg/mL in 2% non-fat dry milk (NFDM, BioRad, Italy) followed by mouse anti-Flag M2 mAb (Sigma, St. Louis, MO, USA) and GAM-HRP (Amresco, Krackeler Scientific, Albany, NY, USA). In-house-produced mouse anti-E7 polyclonal Ab and anti-E7 mAb (Zymed, ThermoFisher Scientific, Europe), diluted 1:500, were used as positive controls. Immune-complexes were revealed by ECL with Super Signal West Pico Chemiluminescent substrate (ThermoFisher Scientific, Europe).

### 4.8. Surface Plasmon Resonance (SPR)

SPR was carried out as described in Accardi et al. [33]. Briefly, the E7 recombinant protein was immobilized to the Biacore CM5 sensor chip using conventional amine coupling. The reaction was performed by injecting E7 at a flow rate of 5 μL/min for 7 min in HBS-P (10 mM Hepes pH 7.4, 0.15 M NaCl, 0.005% (v/v) surfactant P20) at 25 °C. For the epitope mapping, the E7 surface was saturated by an injection of the purified scFv32 at a flow rate of 10 μL/min. Then, 40 μL of the purified scFv51 were injected at 500 nM, followed by the scFv43M2 at the same concentration.

### 4.9. Computational Analyses

Theoretical CDR3 net charge and total CDR charge were calculated, according to Rabia et al. [26] and using the free tool by Kozlowski [32].

TAP analysis was performed using the tool described in Raybould et al. [27] and which is freely available at http://opig.stats.ox.ac.uk/webapps/sabdab-sabpred/TAP.php.

## 5. Conclusions

We performed a theoretical validation of several scFvs against the 16E6 and 16E7 oncoproteins to be used as intrabodies for the treatment of HPV16-associated lesions, and highlighted some characteristics supporting their therapeutic development. Molecular engineering could help improve and tailor specific scFv properties according to the therapeutic needs, even in the direction of a personalized medicine.

The opportunity of using purified scFv proteins directly as therapeutic tools is intriguing as it embodies a very safe delivery system, free from the critical issue of exogenous DNA being internalized and expressed by the cells. However, protein delivery may be less efficient because self-limiting and requiring the continuous administration of the therapeutic molecules, in turn, involves the production of large amounts of the molecule in Good Manufacturing Practice conditions.

## Figures and Tables

**Table 1:** Complementarity-determining Regions (CDRs) of the Anti-16E7 and -16E6 ScFvs.

scFv	CDR1-IMGT (27–38)	CDR2-IMGT (56–65)	CDR3–IMGT (105–117)	CDR1-IMGT (27–38)	CDR2-IMGT (56–65)	CDR3–IMGT (105–117)
anti-16E7		VH (aa)			VL (aa)	
scFv9	GFTF…SSYA	ISGS..GGST	ARGVGAFRPYRKHE	SLR……SYY	GK…….N	NSSPFE..HNLVV
scFv32	GFTF….SSYA	ISGS..GGST	AKQLHK…TLFDY	SLR……SYY	GK…….N	NSSPNK..ANPVV
scFv43M2	GFTF….SSYA	ISGS..GGST	AKVRR….RFDY	QSVS…..SSY	GA…….S	QQRHG….NPAT
scFv51	GFTF….SSYA	ISGS..GGST	AKHLK….GFDY	SLR……SYY	GK…….N	NSSLQH..PPRVV
anti-16E6		VL (aa)			VH (aa)	
scFvI7	QDV……GTA	WA…….S	QQYSS….YPYT	GYTF….TSHW	INPS..NGRT	ARYDG….YFDY

The aa sequences of the VH and VL CDRs of the anti-16E7 and -16E6 scFvs are shown. The aa positions indicated are according to the IMGT unique numbering, which provides standardized limits for the CDRs (CDR1-IMGT: 27 to 38, CDR2-IMGT: 56 to 65 and CDR3-IMGT: 105 to 117). Gaps, indicated by dots represent unoccupied positions. Note that the 14 aa length of the scFv9 VH-CDR3 exceeds the most common length for this region, which is up to 13 aa; therefore, the additional position 112.1 was created between positions 111 and 112.

**Table 2:** Analysis of the ScFv CDRs Net Charge.

**Anti-16E7 ScFvs**	**VH CDR3 Net Charge**	**VL CDR3 Net Charge**	**Total CDR Net Charge**
scFv9	+3.1 (+3.0)	−0.9 (−1.0)	+4.2 (+4.0)
scFv32	+1.1 (+1.0)	+1.0 (+1.0)	+4.1 (+3.9)
scFv43M2	+3.0 (+3.0)	+1.1 (+1.0)	+4.1 (+4.0)
scFv51	+1.1 (+1.0)	+1.1 (+1.0)	+4.2 (3.9)
**Anti-16E6 scFvs**	**VL CDR3 Net Charge**	**VH CDR3 Net Charge**	**Total CDR Net Charge**
scFvI7	0.0 (0.0)	−1.0 (−1.0)	−0.9 (−1.0)

The theoretical net charges of the scFv VH and VL CDR3 were considered separately, and the entire set of six CDRs were calculated by adding the negative charges of glutammate (E, −1) and aspartate (D, −1), and the positive charges of arginine (R, +1), lysine (K, +1) and histidine (H, + 0.1) or using the free tool by Kozlowski LP (2016), IPC-Isoelectric point Calculator with Biology Direct 11: 55 (in brackets, the values calculated at pH 7.4) [32].

**Table 3:** Therapeutic Antibody Profiling of ScFv Candidates.

TAP Metrics	scFv32	scFv43M2	scFv51
CDR length	47	44	45
CDR vicinity PSH score	133.6982	119.791	134.2631
CDR vicinity PPC score	0.4761	3.1258	0.4023
CDR vicinity PNC score	0.0	0.0	0.0
sFvCSP score	2.1	12.4	2.31

Comparison among the anti-16E7 scFvs using the TAP computational tool by Raybould et al. [27] for evaluation of possible therapeutic development. Green boxes indicate a good agreement of the scores with those of post-phase I clinical-stage antibody therapeutics (CSTs); the amber box indicates a score that is less represented in the metric distribution of the 242 antibody therapeutics, considered for setting the parameters. PSH = Patches of Surface Hydrophobicity Metric; PPC = Patches of Positive Charge Metric; PNC = Patches of Negative Charge Metric; SFvCSP = Structural Fv Charge Symmetry Parameter. Results are available at the following links: http://opig.stats.ox.ac.uk/webapps/newsabdab/sabpred/tap_results/20200502_0570079 for scFv32; http://opig.stats.ox.ac.uk/webapps/newsabdab/sabpred/tap_results/20200502_0199339 for scFv43M2; http://opig.stats.ox.ac.uk/webapps/newsabdab/sabpred/tap_results/20200502_0278604 for scFv51 (accessed on 1 June 2020).
